# Metabolic-immune crosstalk in myocardial infarction: RLF and SMCHD1 identified as causal therapeutic targets via integrated lactylation-MR analysis

**DOI:** 10.3389/fcell.2025.1653551

**Published:** 2025-10-13

**Authors:** Juli Tang, Lingyan Yuan, Yong-Li Han

**Affiliations:** ^1^ College of Life Sciences, Shanghai Normal University, Shanghai, China; ^2^ Department of Kinesiology, Institute of Physical Education, Shanghai Normal University, Shanghai, China; ^3^ Acupuncture Department, The First Affiliated Hospital of Henan University of CM, Zhengzhou, Henan, China

**Keywords:** lactylation, mendelian randomization, myocardial infarction, immune cell infiltration, RLF, SMCHD1

## Abstract

**Background:**

The diagnosis of Myocardial Infarction (MI) requires the discovery of specific diagnostic biomarkers beyond high-sensitivity cardiac troponins. To identify causal MI-associated genes regulated by lactylation modification and elucidate their roles in metabolic-immune dysregulation.

**Methods:**

This multi-omics study combined bioinformatic analyses of human MI datasets (GSE60993/GSE61144/GSE66360) with experimental validation to investigate lactylation-related genes (LRGs). Differential expression analysis (limma, P < 0.05, |log_2_FC|>0.585) identified 571 Differentially Expressed Genes (DEGs), which intersected with 2,051 curated lactylation-related genes (LRGs) (PubMed/GeneCards) yielding 56 lactylation-associated DEGs. Mendelian randomization (MR) utilized genetic instruments (P < 5 × 10^−6^) from Gene eQTL and three MI-GWAS cohorts (43,676 cases/128,199 controls), employing inverse-variance weighted (IVW) regression with sensitivity analyses (MR-Egger/weighted median). Functional enrichment (clusterProfiler) of the 56 DEGs examined GO/KEGG terms (FDR P < 0.05), supplemented by Gene Set Variation Analysis (GSVA) of Rearranged L-myc fusion (RLF) and Structural Maintenance of Chromosomes Hinge Domain Containing 1 (SMCHD1) expression strata and CIBERSORT-based immune infiltration assessment. Experimental validation involved LAD ligation-induced MI modeling in C57BL/6 mice, with RLF/SMCHD1 expression quantified via qPCR and Western blot.

**Results:**

Integrated transcriptomic analysis of three GEO datasets (73 MI patients, 67 controls) identified 571 DEGs. Cross-referencing these DEGs with 2,051 LRGs yielded 56 Lactylation-associated DEGs. MR analysis using 42,699 instrumental SNPs established RLF (AUC = 0.823) and SMCHD1 (AUC = 0.809) as causal risk genes that were significantly elevated in MI patients. Functional enrichment implicated both genes in metabolic dysregulation (nucleotide metabolism, HIF-1/MAPK signaling) and necroptosis. Immune profiling revealed increased monocytes, neutrophils, and activated CD4^+^ T cells within MI tissues, all positively correlated with RLF and SMCHD1 expression. Conversely, reduced CD8^+^ T cell infiltration correlated negatively with RLF expression. Independent validation confirmed significant RLF upregulation in MI. Quantitative analyses revealed significant increases in RLF and SMCHD1 expression—at both transcriptional (mRNA) and translational (protein) levels—in MI-induced mice relative to sham controls.

**Conclusion:**

This study pioneers the integration of lactylation modification with MR analysis for MI, establishing RLF and SMCHD1 as causal diagnostic biomarkers. Their dual roles in promoting metabolic dysregulation and pro-inflammatory immune infiltration position them as promising therapeutic targets for MI intervention.

## 1 Introduction

Globally, MI accounts for an estimated annual death toll exceeding 7 million people (Tsao et al., 2022; Wang et al., 2021). Despite advances in coronary revascularization and pharmacotherapy, early MI diagnosis relies predominantly on high-sensitivity cardiac troponins (hs-cTnT/I). These biomarkers suffer from limited specificity, which often delays effective risk stratification and exacerbates post-MI complications (Collet et al., 2021). Growing evidence implicates metabolic-immune dysregulation as a key driver of these complications by promoting fatal cardiac remodeling. Critically, the molecular interplay between metabolic reprogramming and immune activation during MI progression remains poorly characterized. Although transcriptomics has identified numerous MI-associated DEGs, their functional significance and causal relationships with clinical phenotypes are largely unvalidated, hindering therapeutic translation.

The escalating significance of post-translational modifications (PTMs) in cardiovascular pathology amplifies this knowledge gap. Lactylation is a metabolite-sensitive PTM that links glycolytic flux to inflammatory responses through the lactate-HIF1α signaling axis. This modification has established roles as a key regulator in oncology and immunology (Fan et al., 2023; Zhu et al., 2024). However, its impact on MI-related genes, particularly those governing metabolic-immune crosstalk, remains largely unexplored.

Growing evidence identifies lactylation-modified proteins as critical orchestrators of MI pathogenesis. Chuanfu et al. (Fan et al., 2023) revealed that lactate induces Snail1 lactylation in cardiac endothelial cells post-myocardial infarction. Furthermore, Zhang et al. (2023) reported that cardiac metabolism directly regulates sarcomere structure and function through α-MHC lactylation modification, thereby influencing cardiac architecture and performance. Although transcriptomic studies of human MI consistently reveal dysregulation of energy metabolism and immune signatures (Perreault et al., 2024), no prior research has integrated lactylation omics with causal inference approaches to identify causal regulators. Traditional DEG analyses cannot establish causal relationships, and independent PTM studies lack robust genetic validation of clinical relevance. Consequently, two critical questions impede progress: (1) Which lactylation-associated DEGs causally contribute to MI risk? (2) How do these genes simultaneously mediate metabolic dysfunction and immune microenvironment remodeling?

MR provides a powerful approach to address these questions by leveraging genetic variants as instrumental variables (Kurilshikov et al., 2019). However, in cardiovascular research, the integration of lactylation proteomics, multi-cohort transcriptomics, and MR remains unrealized. This methodological gap critically impedes therapeutic discovery. Specifically, effective MI interventions require targets that are both causally supported by genetics and exert dual regulatory functions in metabolic-immune pathways—attributes unverifiable through single-omics strategies. Without integrated frameworks, the diagnostic and therapeutic potential of lactylation-associated differentially expressed genes (lactylation-DEGs) remains untapped.

To address these gaps, we developed an integrative lactylation-MR framework, analyzing three MI transcriptomic cohorts (totaling 73 patients and 67 controls). We identified causal MI-risk genes by integrating lactylation proteomics, DEG screening, and MR analysis, with experimental validation of their roles in metabolic-immune dysregulation and immune microenvironment dynamics. This study establishes RLF and SMCHD1 as lactylation-modified causal regulators of MI pathogenesis, demonstrating their diagnostic and therapeutic potential.

## 2 Materials and methods

### 2.1 Data collection

Human gene expression datasets matching the search terms “myocardial infarction” and “*Homo sapiens*” were retrieved from the Gene Expression Omnibus (GEO) database. Inclusion criteria required: (1) ≥10 samples per cohort; (2) ≥5 MI patients and 5 healthy controls; (3) absence of chemical/genetic interventions; (4) availability of raw data or normalized expression matrices. Three datasets (GSE60993, GSE61144, GSE66360) met these criteria with a total of 73 MI patients and 67 controls (Table 1).

**TABLE 1:** Characteristics of the three GEO datasets.

GSE ID	Samples	Tissues	Platform	Experiment type	Last update date
GSE60993	17 cases and 7 control	peripheral blood	GPL6884	Array	18 Oct 2022
GSE61144	7 cases and 10 control	peripheral blood	GPL6106	Array	18 Oct 2022
GSE66360	49 cases and 50 control	Circulating endothelial cells	GPL570	Array	25 Mar 2019

### 2.2 DEG identification

Data were analyzed in R (v4.4.2) using the limma package for background correction and quantile normalization. Batch effects were adjusted via Principal Component Analysis (PCA). Differential expression analysis employed a linear model with empirical Bayes moderation (P < 0.05, |log_2_FC|>0.585), identifying 571 DEGs (Supplementary Table S1).

### 2.3 LRGs

LRGs were systematically curated from existing literature and public databases, such as PubMed (https://pubmed.ncbi.nlm.nih.gov/) and GeneCards (https://www.genecards.org/), utilizing the search term “lactylation” (Cheng et al., 2023; Huang et al., 2024; Li et al., 2025; Peng et al., 2024; Sheng et al., 2024; Sun et al., 2024; Zhang et al., 2023; Zhao et al., 2025). This process resulted in the identification of 2,051 LRGs, as detailed in Supplementary Table S2. Subsequent intersection analysis between differentially expressed genes (DEGs) and LRGs revealed 56 lactylation-associated DEGs, which are presented in Supplementary Table S3.

### 2.4 MR analysis

Instrumental variables (IVs) were selected as genetic variants significantly associated with lactylation-associated DEG expression (P < 5 × 10^−6^) from the Gene eQTL consortium. Linkage disequilibrium (LD) clumping ensured IV independence (r^2^ < 0.001, distance = 10,000 kb). IVs with F-statistics<10 were excluded as weak instruments, yielding 42,699 SNPs. For outcome data, MI genome-wide association study (GWAS) summary statistics were obtained from: ebi-a-GCST011365 (14,825 cases/44,000 controls, European ancestry) (Hartiala et al., 2021), ieu-a-798 (43,676 cases/128,199 controls, European ancestry) (Nikpay et al., 2015), and ebi-a-GCST90018877 (20,917 cases/461,823 controls, European ancestry) (Sakaue et al., 2021). Primary analysis used IVW regression. Sensitivity analyses employed MR-Egger, weighted median, and simple mode methods. Pleiotropy was assessed via MR-Egger intercept (P > 0.05 indicating robustness).

### 2.5 Functional Enrichment

Functional enrichment analysis of the 56 lactylation-associated DEGs was conducted with the R package clusterProfiler (v4.0), examining Gene Ontology (GO) terms (Biological Process, Molecular Function, Cellular Component) and Kyoto Encyclopedia of Genes and Genomes (KEGG) pathways. Significance was defined as FDR-adjusted P < 0.05. GSVA assessed pathway activity differences between RLF/SMCHD1 high-expression (≥70th percentile) and low-expression (≤30th percentile) groups, consistent with the original study’s stratification.

### 2.6 Immune cell infiltration

Immune cell fractions in MI tissues were quantified via CIBERSORT (v1.03) using the default LM22 signature matrix. Spearman’s rank correlation analysis assessed associations between log_2_-transformed RLF/SMCHD1 expression levels and the proportions of 22 immune cell subsets, with statistical significance defined at P < 0.05.

### 2.7 MI modeling—LAD surgery

Twenty 8-week-old male C57BL/6 mice, weighing 20–25 g, were randomly divided into the MI group (n = 10) and the Sham group (n = 10). Mice were housed in a quiet, temperature-controlled (22 °C–24 °C) environment with a 12-h light/dark cycle. The C57BL/6 mice were anesthetized with 2% isoflurane. A thoracotomy was performed at the fourth intercostal space to expose the heart. The left anterior descending coronary artery (LAD) was ligated approximately 3 mm distal to its origin using a 6–0 silk suture. The suture needle was inserted to a depth of approximately 1.5 mm. Successful occlusion was confirmed by observing a pale discoloration of the myocardial tissue distal to the ligation site. For the sham group, the left anterior descending coronary artery was cannulated without ligation, ensuring that all other surgical procedures were performed uniformly. Finally, the thoracic cavity was closed by suturing the muscle layers sequentially (Chai et al., 2023).

### 2.8 Quantitative real-time PCR

Total RNA was extracted from plasma samples using TRIzol reagent (Invitrogen, Cat# 15596026CN). For each sample, 1 µg of total RNA was reverse transcribed into cDNA using Hifair® III 1st Strand cDNA Synthesis SuperMix (Yeasen, Cat# 11141ES60). Quantitative real-time PCR (qPCR) was subsequently performed using Power SYBR® Green PCR Master Mix (TaKaRa, Cat# RR820A), following the manufacturer’s instructions. The primer sequences used were as follows: LRF: Forward: 5′-CTGTTGCTAAAGGAAATCTGTGC-3′; Reverse: 5′-ACAGTTCTGTAATGGCGAATCAG-3′. SMCHD1: Forward: 5′-GATGGCCTTGACAGCTCAAAC-3′; Reverse: 5′-CGCCAAGTAAAACACAGATCCTT-3′. β-actin (internal control): Forward: 5′-GGCTGTATTCCCCTCCATCG-3′; Reverse: 5′-CCAGTTGGTAACAATGCCATGT-3′. Relative mRNA expression levels were calculated using the 2^−ΔΔCT^ method and normalized to β-actin.

### 2.9 Western blotting

Mouse serum samples were analyzed for RLF and SMCHD1 protein expression by Western blotting. Equal amounts of total protein from each sample were separated by SDS-PAGE and subsequently transferred onto PVDF membranes. After blocking with 5% non-fat milk for 1 h at room temperature, the membranes were incubated overnight at 4 °C with the following primary antibodies: anti-RLF (Abcam, Cat# ab115011), anti-SMCHD1 (Proteintech, Cat# 25589-1-AP), and anti-Transferrin (Proteintech, Cat# 17435-1-AP). Following extensive washing, the membranes were incubated with an appropriate horseradish peroxidase (HRP)-conjugated secondary antibody for 1 h at room temperature. Protein bands were visualized using enhanced chemiluminescence (ECL) substrate and detected/analyzed using a gel imaging system.

### 2.10 Statistical methods

All data are expressed as mean ± standard deviation (SD). Statistical comparisons between Sham and MI groups were performed using unpaired two-tailed Student's t-tests after confirming normality (Shapiro-Wilk test, P > 0.05) and homogeneity of variance (Levene’s test, P > 0.05). For non-normally distributed data, the Mann-Whitney U test was applied. Significance was defined at P < 0.05. All analyses were conducted in SPSS 26.0 (IBM Corp.) and visualized using GraphPad Prism 9.0. Significance thresholds were defined as follows: *** P < 0.001, ** P < 0.01, * P < 0.05, and ns (not significant) for P ≥ 0.05.

## 3 Results

### 3.1 Data integration and DEG screening

Three MI microarray datasets were retrieved from the Gene Expression Omnibus (GEO) database as the experimental cohort, collectively comprising 73 MI patients and 67 healthy controls. Table 1 details the included datasets.

**FIGURE 1:**
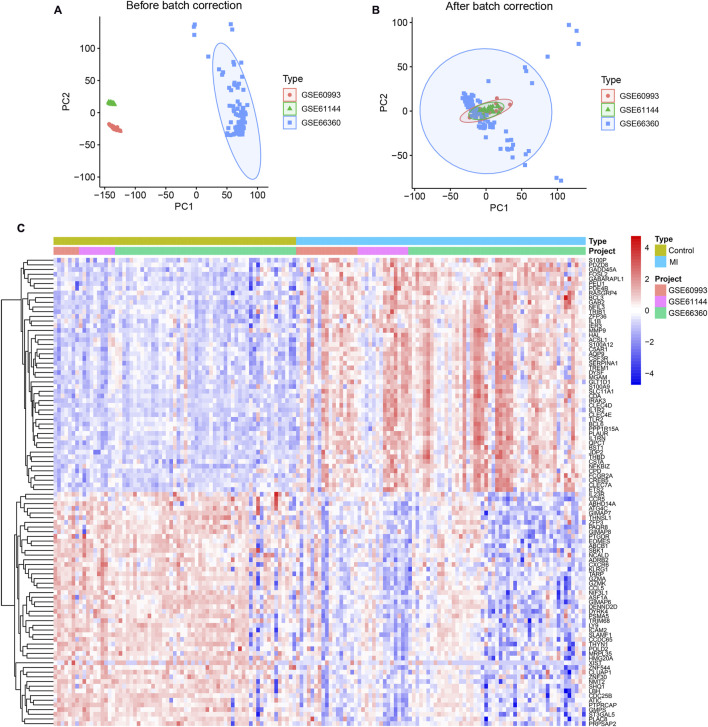
Integrated Transcriptomic Profiling of MI Reveals Differential Expression. **(A)** Uncorrected principal component analysis (PCA) of three transcriptomic datasets (GSE60993, GSE61144, GSE66360) reveals pronounced batch effects. **(B)** Uniform sample distribution following PCA-based batch effect correction, indicating effective harmonization. **(C)** Heatmap of the top 50 DEGs (|log_2_FC|>0.585, P < 0.05) hierarchically clustering MI patients (n = 73) versus controls (n = 67).

Expression values were processed and merged using R (v4.4.2), with batch effects corrected via PCA. Figure 1A illustrates pronounced batch effects across datasets, while Figure 1B demonstrates achieved homogeneity post-correction. Differential expression analysis ranked genes by ascending P-values (lower values indicating higher confidence), identifying 571 significant DEGs (Supplementary Table S1). A heatmap of the top 50 DEGs by statistical significance is shown in Figure 1C.

### 3.2 Identification of Lactylation-associated

**FIGURE 2:**
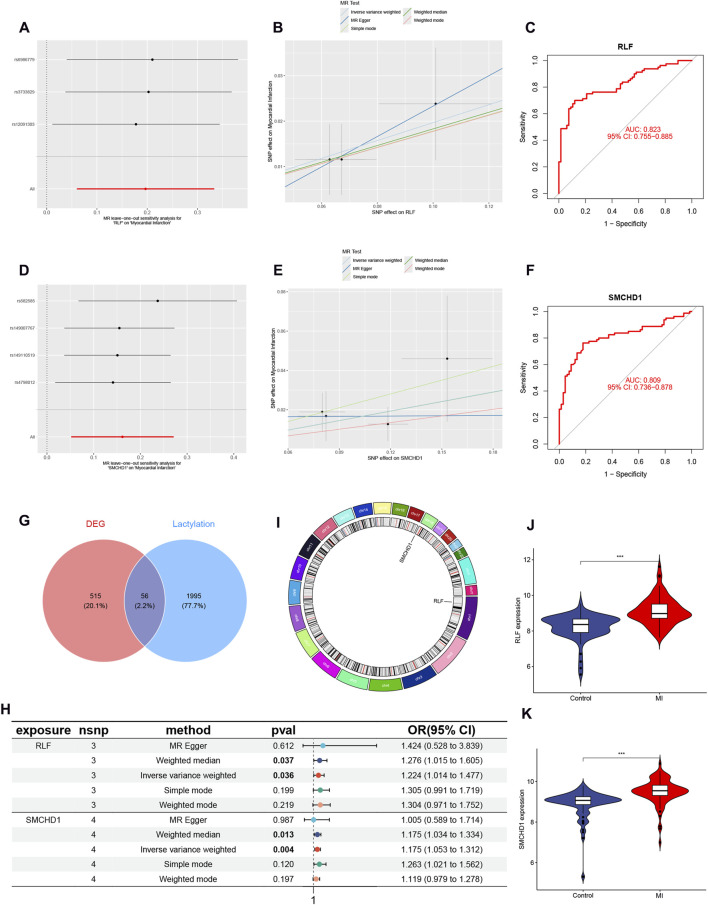
Identification of Lactylation-Associated Causal Genes in MI. **(A)** Leave-one-out sensitivity analysis for RLF in MI GWAS. **(B)** Genetic association scatter plot for RLF. **(C)** ROC curve validating RLF diagnostic performance (AUC = 0.823). **(D)** Leave-one-out sensitivity analysis for SMCHD1. **(E)** Genetic association scatter plot for SMCHD1. **(F)** ROC curve for SMCHD1 diagnosis (AUC = 0.809). **(G)** Venn diagram intersecting 571 MI-associated DEGs and 2,051 LRGs, identifying 56 lactylation-associated DEGs. **(H)** Forest plot of MR results for prioritized genes (RLF and SMCHD1). **(I)** Circos plot displaying genomic localization (RLF: chr1; SMCHD1: chr18). **(J)** Violin plot of RLF expression in MI vs. controls. **(K)** Violin plot of SMCHD1 expression in MI vs. controls. (∗ P < 0.05, ∗∗ P < 0.01, ∗∗∗ P < 0.001).

A total of 2,051 LRGs were curated from literature and databases (Supplementary Table S2). Intersection analysis between DEGs and LRGs identified 56 overlapping genes, designated as lactylation-associated DEGs (Figure 2G; full list in Supplementary Table S3). Through MR analysis under three predefined screening criteria (Supplementary Table S7), we established RLF and SMCHD1 as causal risk genes significantly elevated in MI patients The key MR analyses—including leave-one-out sensitivity (Figures 2A,D), genetic association scatter plots (Figures 2B,E), and the forest plot of the combined IVW estimates (Figure 2H)—visually confirm the robust causal effects of RLF and SMCHD1 on MI risk. RLF is localized to chromosome 1 and SMCHD1 to chromosome 18 (Figure 2I). Receiver operating characteristic (ROC) curves demonstrated high diagnostic accuracy for both genes (RLF: AUC = 0.823, Figure 2C; SMCHD1: AUC = 0.809; Figure 2F) in discriminating MI patients from controls.

### 3.3 Functional enrichment of lactylation-DEGs

**FIGURE 3:**
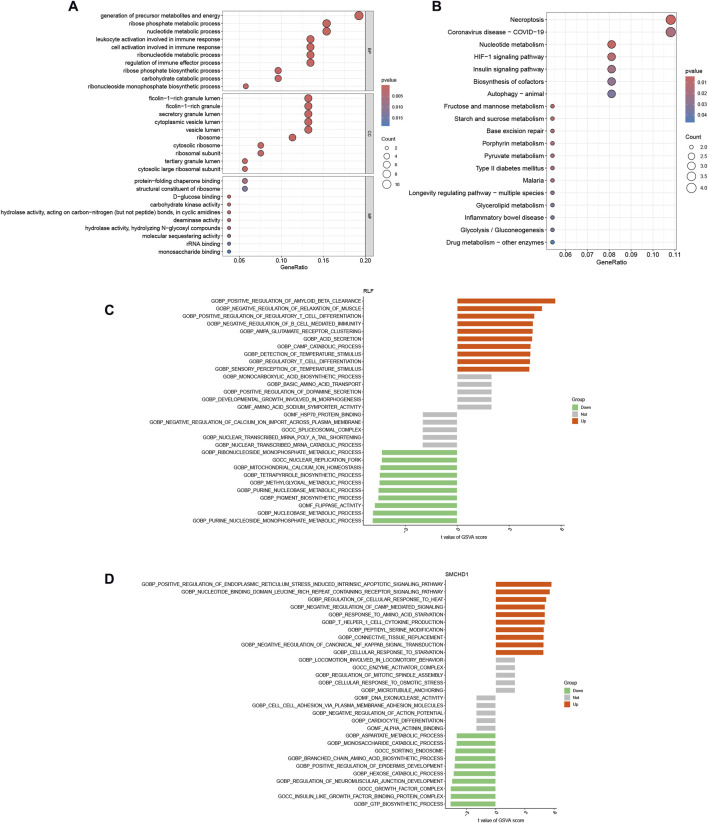
Functional Enrichment of Lactylation-DEGs. **(A)** Gene Ontology (GO) enrichment of 56 lactylation-DEGs. **(B)** KEGG pathway enrichment of 56 lactylation-DEGs. **(C)** GSVA of RLF high-vs. low-expression groups. **(D)** GSVA of SMCHD1 high-vs. low-expression groups.

Functional enrichment analysis of the 56 lactylation-modified differentially expressed genes (DEGs) was conducted using GO and KEGG pathways (Figures 3A,B). GO analysis demonstrated significant enrichment in biological processes including precursor metabolite and energy generation, ribose-phosphate metabolism, leukocyte/cell activation during immune responses, and nucleotide metabolism (Supplementary Table S4). KEGG analysis revealed primary associations with necroptosis, nucleotide metabolism, and the HIF-1 signaling pathway (Supplementary Table S5). GSVA showed RLF-high cohorts exhibited significant upregulation of thyroid cancer, retinol metabolism, dorsoventral axis formation, melanoma, type II diabetes, cytochrome P450-mediated xenobiotic metabolism, neuroactive ligand-receptor interaction, MAPK signaling, tryptophan metabolism, and renin-angiotensin system pathways, alongside downregulation of propanoate metabolism, proteasome function, protein export, peroxisome activity, pentose phosphate pathway, purine metabolism, chondroitin sulfate glycosaminoglycan biosynthesis, primary immunodeficiency, N-glycan biosynthesis, and homologous recombination (Figure 3C). For SMCHD1-high cohorts, significantly upregulated pathways included apoptosis, B-cell receptor signaling, RIG-I-like receptor signaling, chemokine signaling, Leishmania infection response, long-term potentiation, NOD-like receptor signaling, natural killer cell cytotoxicity, Toll-like receptor signaling, and neurotrophin signaling, whereas downregulated pathways comprised ECM-receptor interaction, olfactory transduction, basal cell carcinoma, glycolysis/gluconeogenesis, glyoxylate and dicarboxylate metabolism, Hedgehog signaling, cysteine and methionine metabolism, cytochrome P450-mediated xenobiotic metabolism, phenylalanine metabolism, and tyrosine metabolism (Figure 3D). Crucially, cytochrome P450-mediated xenobiotic metabolism displayed inverse regulation—upregulated in RLF-high cohorts but downregulated in SMCHD1-high cohorts.

### 3.4 Assessment of immune cell infiltration in MI

**FIGURE 4:**
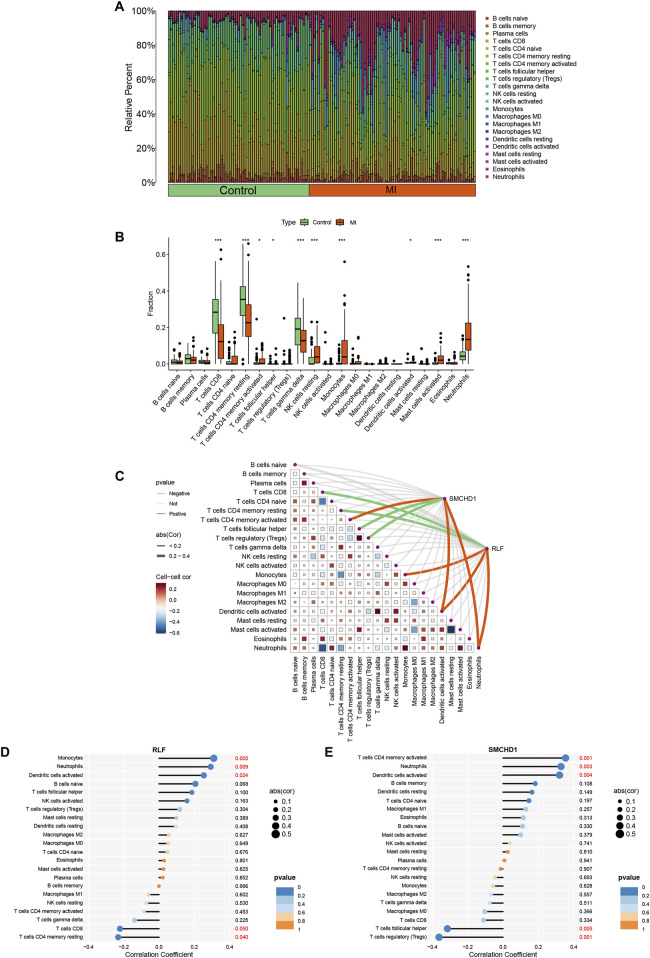
Analysis of immune cell infiltration in MI. **(A)** Stacked histogram illustrating immune cell proportions across MI and control groups. **(B)** Box plot demonstrating differential infiltration levels of 22 immune cell types between MI and control groups. **(C)** Spearman correlation heatmap depicting associations between RLF/SMCHD1 expression and immune cell subsets. **(D)** Lollipop plot visualizing correlations between 22 immune cell types and RLF expression. **(E)** Lollipop plot visualizing correlations between 22 immune cell types and SMCHD1 expression. (∗ P < 0.05, ∗∗ P < 0.01, ∗∗∗ P < 0.001).

Functional and pathway analyses of target genes in MI revealed close associations with metabolic and immune processes. Consequently, we employed the CIBERSORT algorithm to infer immune cell signatures and explore correlations between MI target genes and immune cell infiltration. Figure 4A illustrates the proportions of 22 immune cell types across all samples. Compared with controls, MI patient samples exhibited significantly elevated levels of activated CD4^+^ memory T cells, resting NK cells, monocytes, activated mast cells, and neutrophils, alongside diminished levels of CD8^+^ T cells, resting CD4^+^ memory T cells, and gamma delta T cells (Figure 4B). Furthermore, correlation analysis with the 22 immune cell types demonstrated that the target gene RLF positively correlated with activated dendritic cells, neutrophils, and monocytes, but negatively correlated with CD8^+^ T cells and resting CD4^+^ memory T cells. SMCHD1 showed positive correlations with activated dendritic cells, neutrophils, and activated CD4^+^ memory T cells, while exhibiting negative correlations with follicular helper T cells and regulatory T cells (Tregs) (Figures 4C–E). Notably, activated dendritic cells and neutrophils were positively correlated with both RLF and SMCHD1.

### 3.5 Differential analysis in validation cohort

**FIGURE 5:**
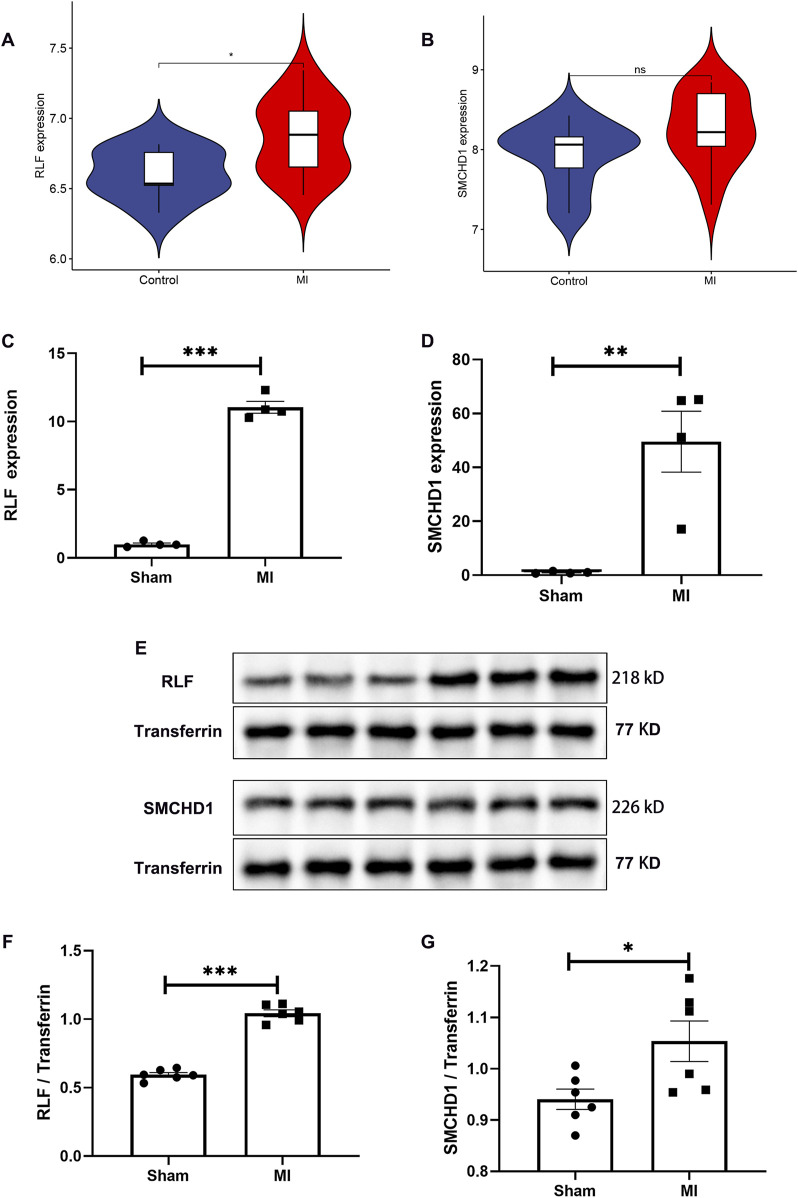
Independent Validation of RLF/SMCHD1 Dysregulation in MI. **(A)** Violin plot illustrating RLF expression levels in MI patients compared to control subjects. **(B)** Violin plot depicting SMCHD1 expression levels in MI patients versus controls. **(C)** Analysis of serum RLF expression in a mouse MI model compared to Sham controls. **(D)** Assessment of serum SMCHD1 expression in a mouse MI model relative to Sham controls. **(E)** Immunoblot analysis of serum proteins in the mouse MI model and Sham controls. **(F)** Evaluation of RLF protein expression in serum from the mouse MI model and Sham controls. **(G)** Analysis of SMCHD1 protein expression in serum from the mouse MI model and Sham controls. (∗ P < 0.05, ∗∗ P < 0.01, ∗∗∗ P < 0.001).

MR analysis revealed significantly elevated expression of RLF and SMCHD1 in the MI group compared with controls (Figures 2J,K). Validation using the GSE61145 cohort confirmed that RLF expression was significantly higher in MI samples than in controls (P < 0.05; Figure 5A), consistent with our MR findings. Although SMCHD1 expression was elevated in the MI group relative to controls, this difference did not reach statistical significance (Figure 5B).

### 3.6 Validation of RLF and SMCHD1 dysregulation in the mouse MI model

In the mouse MI model, serum RLF expression was significantly elevated at both mRNA and protein levels compared with sham controls (P < 0.0001; Figures 5C,F). WB analysis confirmed increased RLF protein expression in MI serum (Figure 5E), with quantitative data showing marked upregulation (Figure 5F). SMCHD1 expression demonstrated upward trends in mRNA (P = 0.0051, Figure 5D) and protein levels (P = 0.0289, Figures 5E,G) in the MI group.

## 4 Discussion

### 4.1 Restatement and innovation of core discoveries

This study pioneers the integration of lactylome analysis with MR to establish RLF and SMCHD1 as causal risk genes for MI. By screening 571 DEGs identified in three MI transcriptomic datasets (73 patients vs. 67 controls) and intersecting them with 2,051 LRGs, we obtained 56 lactylation-associated DEGs. Subsequent MR analysis (utilizing 42,699 instrumental SNPs with F-statistic>10) confirmed the causal effects and diagnostic value of RLF (AUC = 0.823) and SMCHD1 (AUC = 0.809). Functional investigations revealed that both genes cooperatively drive metabolic dysregulation (affecting nucleotide metabolism and HIF-1/MAPK signaling) and immune cell infiltration (characterized by monocyte/neutrophil, activation and CD8^+^ T-cell suppression), highlighting the central role of lactylation in metabolic-immune crosstalk. This multi-omics causal inference framework provides a novel paradigm for cardiovascular target discovery.

### 4.2 The mechanism and pathological significance of RLF

RLF which encodes a zinc finger protein of the Zn-15 family (also known as ZN-15L or ZNF292L), plays a role in regulating gene transcription by binding to specific DNA sequences through its zinc finger domain. It can either activate or repress the expression of target genes, thereby influencing fundamental cellular processes such as proliferation, differentiation, and apoptosis. Dysregulation of RLF expression or mutations in this gene can disrupt cell cycle control and may contribute to tumorigenesis. Additionally, altered RLF expression levels have been reported in neurodegenerative conditions, suggesting potential roles in neuroprotection and repair. RLF exacerbates MI injury through two distinct mechanisms: (1) Metabolic dysregulation: RLF activates the MAPK signaling pathway, which is implicated in thyroid cancer and retinol metabolism, as well as cytochrome P450-mediated xenobiotic metabolism, leading to energy stress and oxidative damage in ischemic myocardium. (2) Immune reprogramming: RLF is positively correlated with monocyte and neutrophil infiltration and negatively correlated with CD8^+^ T cells and resting CD4^+^ T cells, fostering a pro-inflammatory microenvironment. Within the cardiovascular system, the functional role of RLF has remained largely undefined. This study is the first to identify RLF as a key node in metabolic-immune crosstalk. Mechanistically, its zinc finger domain may bind to lactylated histones such as H3K9la, amplifying the HIF-1α-mediated glycolysis-inflammation positive feedback loop, thereby accelerating necroptosis within the infarct zone. This is supported by enrichment analysis of the necroptosis pathway.

### 4.3 The mechanism of SMCHD1 and its synergy with RLF

SMCHD1 is a large, multi-domain protein belonging to the SMC superfamily. As a key epigenetic modifier essential for normal development, SMCHD1 participates in gene silencing (Dong et al., 2023). Missense mutations in the SMCHD1 gene impair its gene-silencing capacity and can underlie Bosma arhinia microphthalmia syndrome (BAMS) (Gendrel et al., 2013). Furthermore, SMCHD1 restricts herpesvirus replication by binding viral lytic origins and inhibiting replication complexes; mutations in SMCHD1 cause facioscapulohumeral muscular dystrophy type 2 (FSHD2) through aberrant gene silencing (Tian et al., 2023). Our GSVA in MI revealed a dual regulatory mechanism mediated by SMCHD1. First, this zinc finger protein promotes pro-inflammatory activation through upregulation of B-cell receptor signaling, Toll-like receptor (TLR)/NOD-like receptor pathways, and neutrophil-mediated cytotoxicity–consistent with its documented role in amplifying innate immune responses (Tian et al., 2023). Second, it demonstrates contrasting metabolic regulation by suppressing cytochrome P450-mediated xenobiotic metabolism (notably through CYP3A4 downregulation), a function that directly opposes RLF’s enhancing effects. This metabolic-immunological duality suggests tissue-specific epigenetic reprogramming, where SMCHD1 simultaneously executes two distinct functions: (1) silencing metabolic genes via chromatin modification, and (2) activating inflammatory transcriptional programs in ischemic cardiomyocytes. The coordinated suppression of metabolic pathways alongside immune activation reveals a novel mechanistic link between cellular energetics and inflammatory responses during ischemic injury.

### 4.4 Clinical translational value of metabolic immune crosstalk

Monocytes exhibit phenotypic plasticity, playing dual roles in the pathology following MI. The necrosis of myocardial tissue leads to the release of damage-associated molecular patterns (DAMPs), such as high mobility group box 1 (HMGB1), which initiate the infiltration of monocytes. These monocytes subsequently differentiate into distinct macrophage subsets: M1 pro-inflammatory (characterized by CCR2+CD86^+^ markers) and M2 reparative (characterized by CX3CR1+CD206+ markers). M1 macrophages exacerbate tissue damage through IL-1β/TNF-α secretion, while M2 counterparts promote fibrotic repair via TGF-β/IL-10 production (Gombozhapova et al., 2017). Our findings indicate that the overexpression of RLF significantly enhances the recruitment of monocytes (r = 0.42, p < 0.01), potentially mediated through the activation of the MAPK pathway and the CCL2/CCR2 axis. In contrast, SMCHD1 exerts an epigenetic suppression on anti-inflammatory genes, such as IL10, thereby delaying the polarization towards the M2 macrophage phenotype (Liu et al., 2016; Nahrendorf et al., 2010). Therapeutically, modulating the RLF-SMCHD1 axis enables stage-specific intervention: RLF inhibition during acute phase attenuates M1 polarization, while SMCHD1 blockade in repair phase facilitates M2-mediated tissue restoration.

Neutrophils exhibit temporally divergent roles in MI pathogenesis. During the early phase (6–24 h post-infarction), neutrophil infiltration exacerbates myocardial injury through NETosis-mediated release of myeloperoxidase (MPO), amplifying oxidative stress. Conversely, a phenotypic transition occurs at 72 h, with neutrophils adopting an N2 reparative subtype characterized by lactoferrin and lipoxin A4 (LXA4) secretion, which facilitates inflammation resolution (Sager et al., 2017). Our findings demonstrate coordinated regulation by RLF and SMCHD1, which synergistically upregulate neutrophil chemokines CXCL1/CXCL8, correlating strongly with infiltration intensity (r > 0.38, p < 0.01). Notably, metabolic crosstalk analysis revealed functional antagonism: RLF activates while SMCHD1 suppresses cytochrome P450 pathways (Figures 3C,D), potentially disrupting redox homeostasis through impaired reactive oxygen species (ROS) clearance. Therapeutic modulation of the RLF-SMCHD1 axis emerges as a promising strategy to mitigate early neutrophil-mediated cytotoxicity while preserving their later reparative functions.

CD8^+^ T lymphocytes exhibit functional dichotomy in MI pathogenesis, with cytotoxic (CD8^+^CD57^+^) and regulatory (CD8^+^CD28^−^) subsets playing opposing roles. Cytotoxic populations exacerbate cardiac injury through IFN-γ and granzyme B secretion, directly inducing cardiomyocyte death and adverse outcomes (Tae Yu et al., 2015). Conversely, regulatory CD8^+^ T cells attenuate inflammatory cascades via PD-1/CTLA-4 checkpoint signaling while promoting tissue repair. Our mechanistic studies demonstrate RLF-mediated suppression of total CD8^+^ T cell infiltration (r = −0.31, P < 0.05; Figure 4C), potentially through MHC-I downregulation that may compromise anti-fibrotic surveillance. Significantly, the hyperactivation of the MAPK pathway driven by RLF transcriptionally upregulates markers of T cell exhaustion, such as TIM-3, thereby preferentially expanding pathogenic CD57^+^ subpopulations (Figure 3). This dual regulatory mechanism positions RLF as a pivotal regulator of CD8^+^ T cell functional polarization within ischemic myocardium.

Resident memory CD4^+^ T cells (Trm) serve as an antigen-specific immunological memory reservoir that rapidly activates and recruits macrophages upon MI recurrence (Dong et al., 2023). Our findings suggest that epigenetic silencing mediated by SMCHD1 may suppress critical survival genes of tissue-resident memory T cells (Trm), such as BCL2, thereby impairing immune memory. Clinically, a decreased frequency of Trm is associated with an increased risk of cardiac rupture following MI, offering a theoretical basis for targeting SMCHD1 in therapeutic interventions.

### 4.5 Research limitations and future directions

Our integrated approach, which combines lactylomic profiling, multi-cohort transcriptomics, and MR analysis, addresses significant limitations inherent in single-omics studies, such as correlative inference bias. However, several challenges remain that require further investigation. From a technical standpoint, the heterogeneity of tissue data sources—such as peripheral blood versus circulating endothelial cells (refer to Table 1)—may obscure myocardial-specific lactylation targets. To address this, future research should utilize single-cell lactylome sequencing to elucidate modification landscapes across various infarct-zone cell subtypes, including CD206^+^ reparative macrophages and cardiac fibroblasts, thereby defining the cell-type-specific functions of RLF/SMCHD1 (Zhu et al., 2024). Regarding population bias, MR analysis relied on European-derived genetic instruments (42,699 SNPs), potentially limiting generalizability due to interethnic linkage disequilibrium variations. Validation in multi-ethnic cohorts (East Asian, African) is essential to confirm target causality. Critical mechanistic gaps persist: (1) The direct lactylation of RLF/SMCHD1 proteins necessitates validation through the use of site-specific antibodies, such as anti-RLF-Kla, in conjunction with mass spectrometry to accurately map the modification sites and assess their dynamics; (2) Temporal regulation of RLF/SMCHD1 across infarction phases (pro-inflammatory acute vs. pro-fibrotic repair) remains uncharacterized. We recommend generating cardiac-specific conditional knockout models for time-resolved transcriptomic and lactylomic profiling to delineate stage-dependent functions.

Theoretically, we propose lactylation as a novel “metabolo-immunological checkpoint”: Lactate accumulation synchronously drives metabolic stress and immune imbalance through RLF/SMCHD1 modification. Clinical translation should be phased: The short-term integration of RLF/SMCHD1 with established biomarkers, such as SAT1 and PYGL5, has the potential to develop lactylation-DEG pathway maps applicable to multicenter diagnostic frameworks. Long-term strategies should develop targeted modulators—designing zinc finger domain inhibitors against RLF and employing lysosome-targeting chimeras (LYTACs) for SMCHD1 degradation.

## 5 Conclusion

This investigation provides an in-depth analysis of MI through advanced bioinformatic and statistical approaches, identifying key genes and pathways with high specificity. We highlight the pivotal roles of immune cells and genetic determinants in MI pathogenesis, with RLF and SMCHD1 emerging as principal molecular targets. These findings not only elucidate the complex molecular mechanisms underlying MI but also open avenues for novel therapeutic interventions. Collectively, our study contributes significantly to the understanding of MI pathophysiology and underscores the paradigm shift toward targeted molecular and immunomodulatory strategies in future therapeutic development.

## Data Availability

The original contributions presented in the study are included in the article/Supplementary Material. Further inquiries can be directed to the corresponding author(s). The publicly available datasets analyzed in this study can be found in online repositories. The names of the repository/repositories and accession number(s) can be found below: (1) Gene Expression Omnibus (GEO): GSE60993: https://www.ncbi.nlm.nih.gov/geo/query/acc.cgi?acc=GSE60993 GSE61144: https://www.ncbi.nlm.nih.gov/geo/query/acc.cgi?acc=GSE61144 GSE66360: https://www.ncbi.nlm.nih.gov/geo/query/acc.cgi?acc=GSE66360 (2) GWAS summary statistics were obtained from the IEU OpenGWAS and GWAS Catalog platforms:ebi-a-GCST011365: https://opengwas.io/datasets/ebi-a-GCST011365ieu-a-798: https://opengwas.io/datasets/ieu-a-798ebi-a-GCST90018877: https://opengwas.io/datasets/ebi-a-GCST90018877 (3) The list of lactylation-related genes (LRGs) was curated from published literature and the GeneCards database (https://www.genecards.org/).
